# Characterizing the contribution of high temperatures to child undernourishment in Sub-Saharan Africa

**DOI:** 10.1038/s41598-020-74942-9

**Published:** 2020-11-02

**Authors:** Rachel E. Baker, Jesse Anttila-Hughes

**Affiliations:** 1 Princeton Environmental Institute, Princeton University, Princeton, NJ USA; 2 Department of Economics, University of San Francisco, San Francisco, CA USA

**Keywords:** Environmental health, Environmental economics

## Abstract

Despite improvements to global economic conditions, child undernourishment has increased in recent years, with approximately 7.5% of children suffering from wasting. Climate change is expected to worsen food insecurity and increase potential threats to nutrition, particularly in low-income and lower-middle income countries where the majority of undernourished children live. We combine anthropometric data for 192,000 children from 30 countries in Sub-Saharan Africa with historical climate data to directly estimate the effect of temperature on key malnutrition outcomes. We first document a strong negative relationship between child weight and average temperature across regions. We then exploit variation in weather conditions to statistically identify the effects of increased temperatures over multiple time scales on child nutrition. Increased temperatures in the month of survey, year leading up to survey and child lifetime lead to meaningful declines in acute measures of child nutrition. We find that the lifetime-scale effects explain most of the region-level negative relationship between weight and temperature, indicating that high temperatures may be a constraint on child nutrition. We use CMIP5 local temperature projections to project the impact of future warming, and find substantial increases in malnutrition depending on location: western Africa would see a 37% increase in the prevalence of wasting by 2100, and central and eastern Africa 25%.

## Introduction

Mounting evidence indicates that high ambient temperatures are a substantial threat to public health, a particular concern given the increased population exposure to heat expected under anthropogenic climate change^1–3^. Methodological advances and expanded data availability have led to more precise identification of these effects across domains^4–6^, ranging from better damage estimates for direct exposure risks such as heat exhaustion and death, to exploring and quantifying indirect influences of heating-driven changes in environmental and socioeconomic factors supporting population health^4,5,7–9^. Many of these damages are particularly concentrated in low-income and lower-middle income countries that are exposed to higher average temperatures, may have lower adaptive capacity, and where livelihoods are more directly dependent on environmental conditions. High temperatures reduce food security by lowering agricultural productivity^10–14^, alter transmission dynamics for a range of diseases^15–19^, increase water scarcity and worsen sanitation outcomes^19,20^, increase the risk of violent conflict^5,21^, and reduce labor productivity, incomes, and economic growth^22,23^.

A consistent finding in this literature is the heightened vulnerability of children^24^, who both have less resilient biological coping mechanisms and are often differentially exposed to excess heating due to behavioral and social reasons such as outdoor play or agricultural chores^25,26^. Exposure to high ambient temperatures leads to a range of acute health problems in children^4,27^, including thermal stress-induced dehydration and heat exhaustion, exposure to changes in disease environment including increased prevalence vector borne and diarrheal diseases^19,27–33^, all culminating in broadly increased mortality risk of high temperature exposure among infants^4,5,34,35^. Excess heating may have negative effects on fetal development, leading to decreased birthweights^36–38^ and altered cohort size and composition through miscarriages, scarring, and differential fertility responses^39–44^.

While the mortality and immediate health effects of heating are increasingly well understood, the child growth and development effects of heat remain understudied, particularly in low-income and lower-middle income countries. Heating is expected to have different implications for child development depending on the scale of exposure. At time scales of days to weeks, high temperatures may directly influence a child’s ability to retain available nutrients through thermal stress-induced appetite loss, increased dehydration, or increased diarrhea, leading to poor absorption of nutrients and calories and acute malnutrition in the form of weight loss^19,27,33,45^. At longer time scales, high temperatures may lower nutritional intake indirectly by reducing total agricultural yields as well as the nutritional value of yields^3,10–12,14,46–48^. If households have insufficient access to food or saved income sufficient to maintain consumption during periods of low yields, reducing household nutritional intake is an unavoidable response^49,50^; evidence suggests that reducing food intake occurs prior to other adaptive measures such as temporary migration, during periods of famine^49^. Reduced nutrition over prolonged periods can lead to chronic malnutrition and stunting, leaving children susceptible to diseases such as malaria, respiratory tract infections, and intestinal infections^51^, as well as ultimately increased mortality^52,53^.

Here we combine a large sample of child anthropometric data from the Demographic and Health Surveys with spatiotemporally-varying climate data from the University of Delaware to quantify the relationship between increased ambient temperatures and child nutrition in Sub-Saharan Africa. We focus on Sub-Saharan Africa for several reasons. Sub-Saharan Africa has high rates of malnutrition as well as a relatively large concentration of the world’s youth, containing 17% of the world’s children under 5 but a third of all undernourished children as of 2015^54^. Second, climate change-driven threats to food security are expected to be particularly severe in Sub-Saharan Africa due to both more damaging shifts in the climate as well as lower baseline food security^3,11,12,46,48^. Finally, the region has unusually rich geolocated health survey data, allowing us to link child health data to specific weather outcomes, generating a sample of over 200,000 children surveyed between 1993–2012 in 30 countries. Our approach allows us to not only explore the cross-sectional average relationship between temperature and child weight measures, but also to apply statistical techniques from the climate impacts literature^5,6,22^ to identify how excess temperature exposure at different time scales influences both acute and chronic nutrition outcomes.

We match each child’s anthropometric data (child weight-for-height, child weight-for-age and child height-for-age) to historical temperature data using their location and time of survey. We calculate average temperature for the month of survey, the 12 months leading up to survey, and the child’s entire lifetime. We then estimate statistical models to capture the average relationship between temperature and child anthropometric measures while controlling for household demographic outcomes and precipitation, as well as a complete set of binary indicator variables (i.e., fixed effects) for the calendar month, year, and subnational administrative region in which each child was surveyed. Doing so allows us to identify the average partial effect of temperature on child nutrition by using differences in temperature exposure for children born in the same administrative region but surveyed at different times, while simultaneously controlling for trends over time^6,22^.

Our approach provides empirical evidence of high ambient temperature’s effects on both classically acute (i.e., weight-based) and chronic (i.e., height-based) measures of child malnutrition. We limit our sample to children between the ages of 1 and 5, excluding infants from our main analysis so as to minimize confounding infant mortality, fertility, and fetal selection effects of temperature, for which we find substantial evidence in the sample (Fig. S22–S24, “Methods”). While our models include controls for precipitation, which is known to also affect child development metrics^55^, we focus on temperature because of its importance in the climate change context^3^ given uncertainty in future precipitation projections^56^.

**Figure 1:**
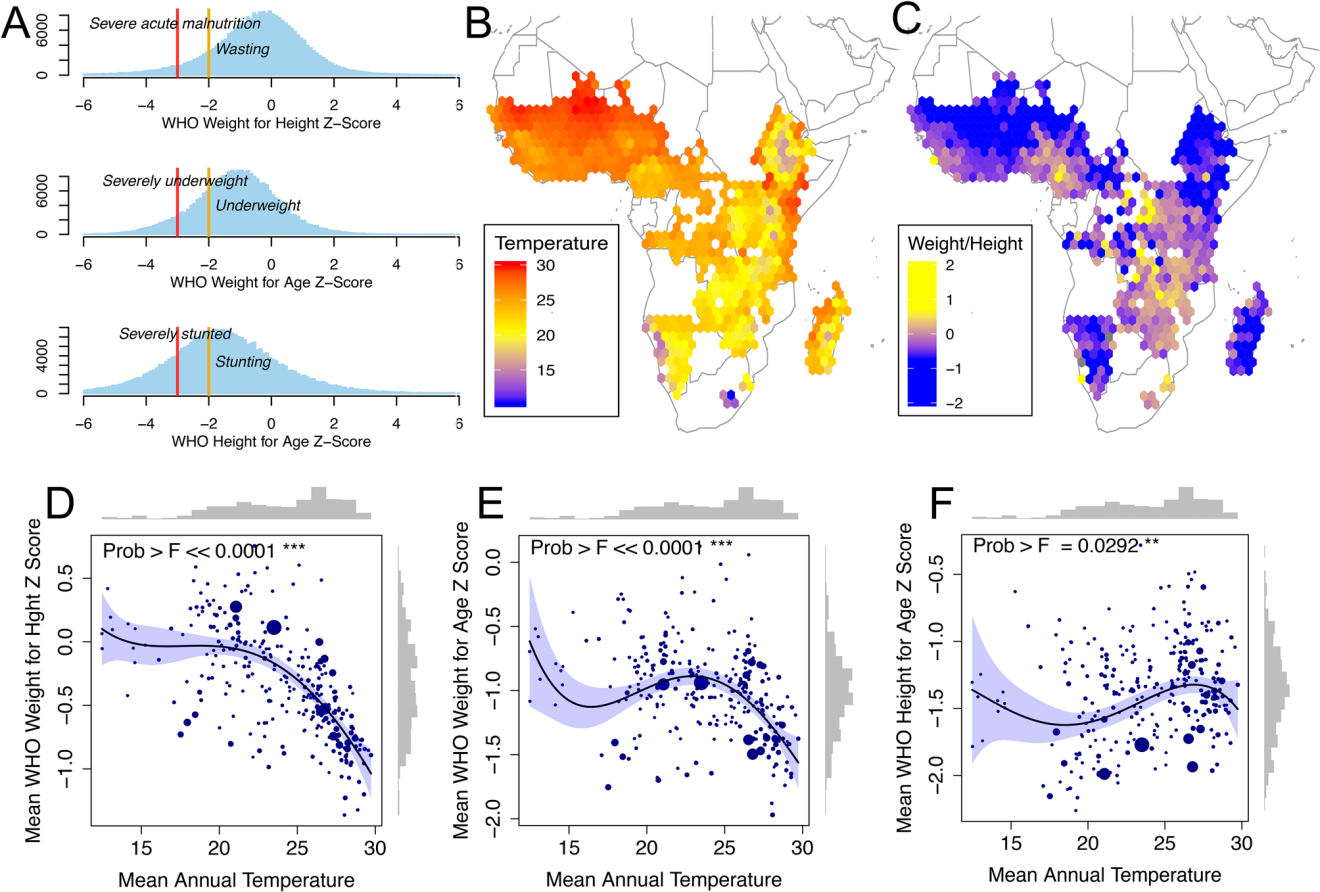
Average temperature and child anthropometric outcomes. (**A**) Distribution of child WHO weight and height measures in sample, weighted using DHS sample weights to be representative at region-by-survey level. WHO wasting, underweight, and stunting thresholds shown for moderate (yellow, − 2$\sigma$) and severe (red, − 3$\sigma$) cases. (**B**) Spatial variation in 50-year average temperature and (**C**) average child weight-for-height for gridded 1.5 * 1.5$^{\circ }$ bins of latitude and longitude. (**D**–**F**) Sub-national regional (admin level 1) correlation between average (50 years) regional temperature and average anthropometric measures for children surveyed in that region. A fourth-order polynomial is fitted to the data, weighted by number of observations (proportional to dot size). The F-statistic for the polynomial fit is shown along with histograms of the distribution of the data.

## Results

Figure 1 presents prima facie evidence of population associations between heating and child anthropometrics. Histograms of individual child-level DHS data in panel A show that median World Health Organization (WHO) standardized^57^ child weight-for-height, weight-for-age and height-for-age in our sample are far below the WHO standards, consistent with documented widespread undernutrition in Sub-Saharan Africa. Panels B and C map long-run average temperature (50 years) and sample average weight-for-height gridded over the spatial extent of our anthropometric dataset, using 1.5 * 1.5$^{\circ }$ bins. Temperatures are generally higher in northern Africa close to the Saharan desert, and cooler in mountainous regions such as in Ethiopia and Lesotho; weight follows a visibly inverse spatial pattern, with Z-Scores ranging from − 2$\sigma$ in the northern-most Sahara and the Namibian desert to + 1$\sigma$ in southern Africa. Panels D–F quantify this relationship in the cross section, plotting average temperature and child outcomes by first subnational administrative region in the data, demonstrating a steep decline in weight with high temperatures. Regions averaging 30 $^{\circ }$C have approximately 1$\sigma$ lower weight-for-height Z-Scores than children in regions with a temperature averaging 20 $^{\circ }$C, or a 0.1$\sigma$ loss per 1 $^{\circ }$C decline. Height measurements, in comparison, do not show as clear of a spatial relationship with temperature, increasing slightly in warm compared to cool areas, before declining in the hottest regions.

**Figure 2:**
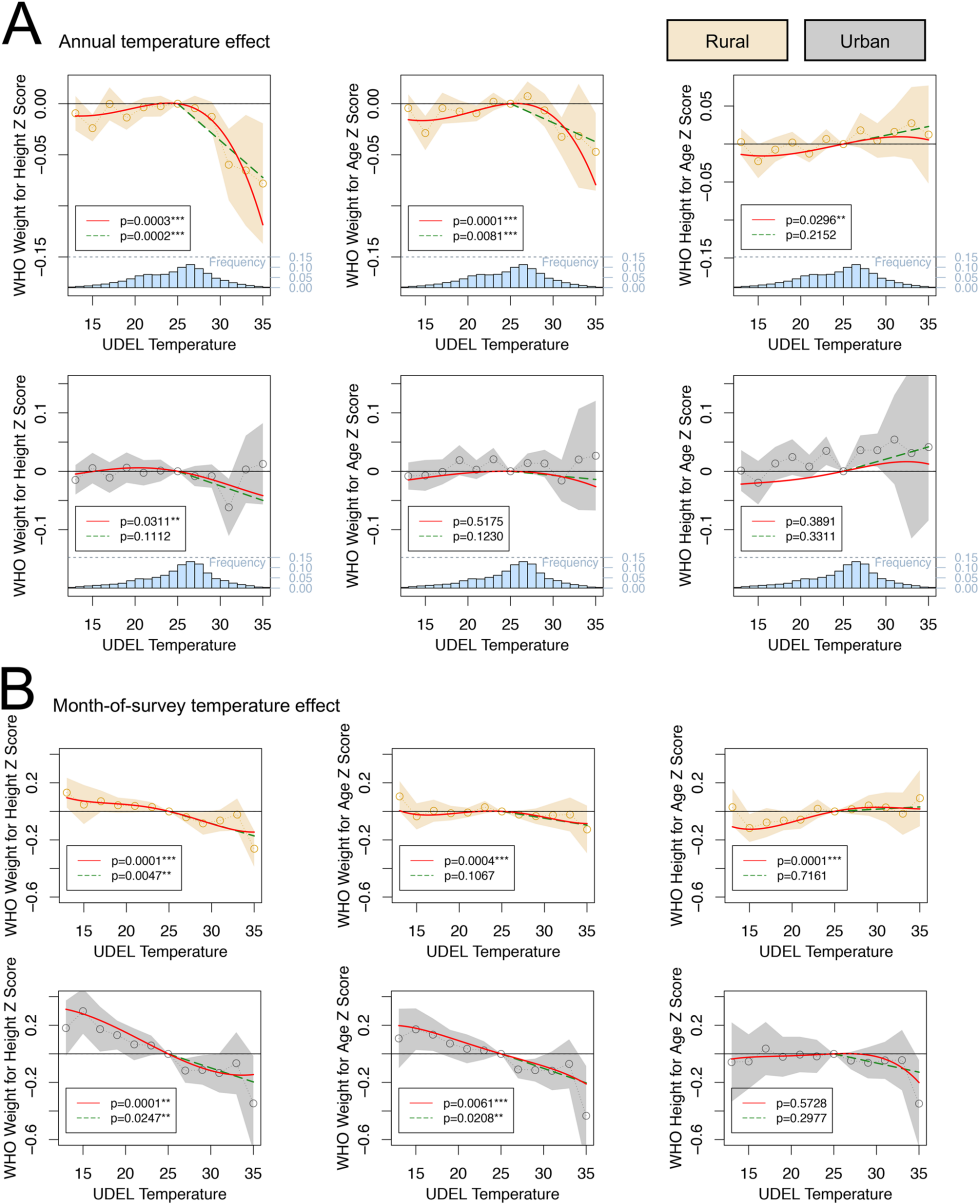
Effects of annual and monthly temperature on child growth. Plots show the marginal effect of a month with a given average temperature occurring in the previous year (**A**) or the month of survey (**B**), relative to the omitted category of a month with an average temperature of 25 $^{\circ }$C. Outcomes from left to right are weight-for-height, weight-for-age and height-for-age Z-scores. Each graph shows three models: a binned model in gold (rural) or gray (urban) shown with confidence intervals; a fourth order polynomial in red; and a degree-month model in green. All models include region, interview year, and interview month fixed effects, as well as controls for precipitation and child and mother demographics. *p* values for the polynomial (joint significance of polynomial terms) and degree-month model are shown and a histogram of the temperature support is at the base of the plot.

While these population associations are meaningful, they are easily confounded by the many other factors associated with average temperature and child outcomes across geographies. To address these issues, we link the anthropometric data on each 1–5 year old child in our study with location-specific weather conditions in a pooled statistical model. We estimate the average relationship between experienced temperatures and child nutritional status over several time periods leading up to the time of survey, controlling for household and regional characteristics that might confound the relationship.

Figure 2 shows results from our main regression model estimating the relationship between child-level outcomes and temperature. Here we estimate the effect of temperature variation over two time scales leading up to the time of child survey: annually in panel A, and monthly in panel B. We divide the sample into rural and urban subsamples and estimate relationships within them to highlight the pronounced difference in average responses between groups, though pooled estimates reveal substantively similar responses (Supplementary Materials Fig. S6, S7). To capture key nonlinearities in responses, estimates are normalized to be relative to a baseline omitted category of 25 $^{\circ }$C average monthly temperature.

We find a significant (*p* $\ll$ 0.01) nonlinear negative relationship between acute child malnutrition and high temperatures that persists across multiple time scales. Increased temperatures during the year leading up to survey (2A) have their strongest effect in rural locations, where weight-for-height and weight-for-age decline sharply with temperatures above 25 $^{\circ }$C. The results imply that a 1 $^{\circ }$C change in annual temperature leads to an approximate 0.08$\sigma$ decline in weight-for-height (summing temperature effects across 12 months in the year). This decline is almost equivalent to the cross-sectional results where we found a 0.1$\sigma$ per 1 $^{\circ }$C gradient (Fig. 1D).

Responses to annual temperature variation in urban areas are comparatively flat. Increased monthly temperatures (2B) meanwhile have little effect in rural areas, but are associated with significant (*p* $\ll$ 0.001) linear decreases in child weight measures in urban locations across the distribution of temperature. These weight losses are also meaningful in size, with a shift in average temperature the month of survey from 25 to 35 $^{\circ }$C causing a 0.2$\sigma$ reduction in weight-for-height. These results echo the documented association between temperature increases and diarrhea, particularly in urban areas where sanitation problems are compounded^19,27,28,30–33^. Using recall data on reported cases of childhood diarrhea by mothers, we find that monthly temperatures are also correlated with diarrhea in these data, but only in urban locations (Supplementary Materials Fig. S10).

**Figure 3:**
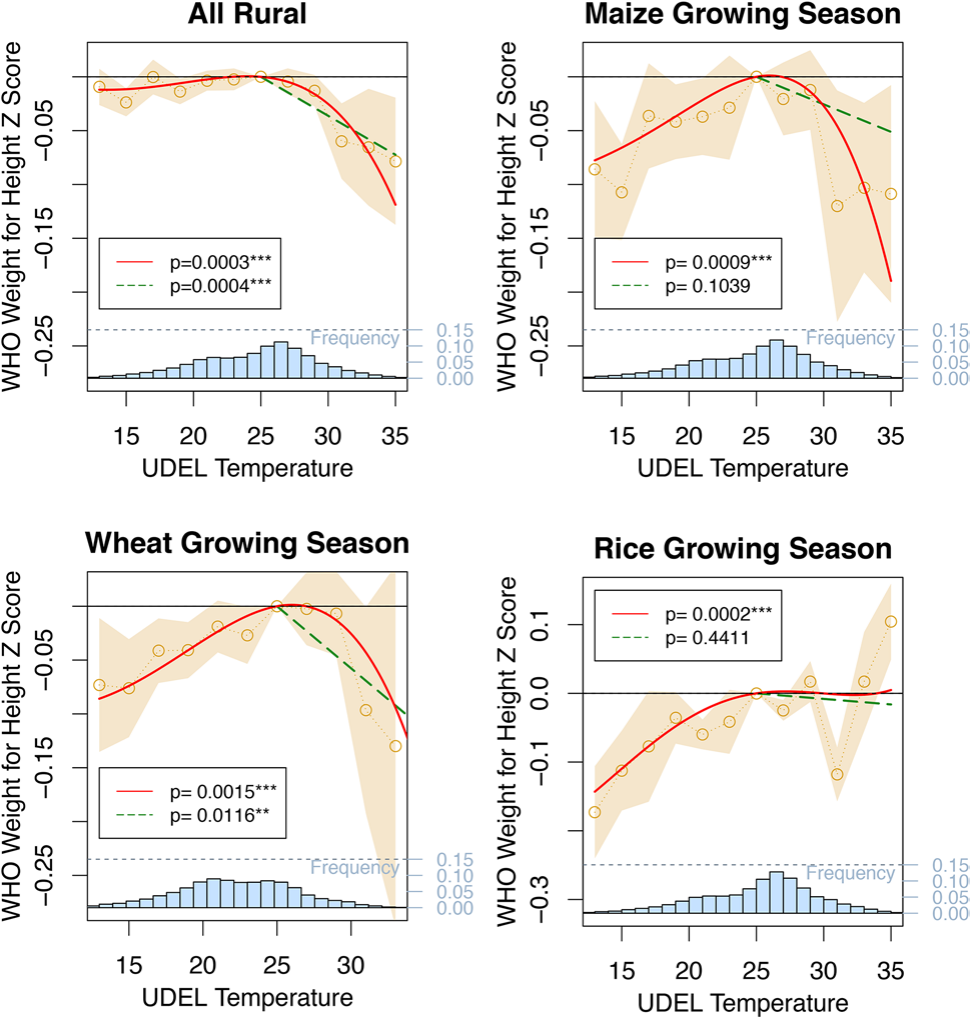
Growing season temperatures on child weight-for-height. The effect of temperatures in the previous year’s growing season on child weight-for-height z-score. The first plot shows for scale the rural result from Fig. 2. The effect of temperatures in the maize, wheat and rice growing season are shown, with the same models and controls as Fig. 2. *p* values for the polynomial (joint significance of polynomial terms) and degree-month model are shown.

We do not see an effect of recent temperatures on chronic measures of malnutrition such as height-for-age and stunting, which may be for several reasons. First, height changes slowly and cumulatively, reflecting total lifetime nutritional conditions more than recent shocks. This likely makes monthly and annual variation too brief to substantially influence height for most children in our sample of $>1$ year olds^52^. Second, the relationship between wasting and stunting in particular, and acute versus chronic malnutrition in general, is known to be complex, and it is not clear that weight loss necessitates height loss in all populations^58^. Lastly, very young children, who are growing most quickly and hence most likely to show short-term height losses, are also at greatest risk of suffering heat-related mortality, as well as reduced cohort size due to lower conception rates and fetal losses^42,43^. This may bias sample composition to healthier, and hence taller, children that are more likely to survive. We document evidence consistent with this effect in the form of counterintuitive positive effects of high temperatures on lifetime height that are driven by high temperatures in very early infancy i.e. under age one (see “Methods” and Fig. S15, S17).

Our annual results in rural areas show a similar form to the nonlinear decrease in crop-yields at higher temperature demonstrated in the climate-agronomy literature^10–14,46,48^. We further test for evidence supporting agriculture as a primary driver in the relationship between temperatures and child weight by estimating the effect of temperatures specifically during agricultural growing seasons (Fig. 3), accounting for the crop types grown in a particular location (“Methods”^59^). We find large effects of temperature on rural children’s weight-for-height in the growing season. Maize and wheat-growing season temperatures lead to markedly more pronounced losses than the annual aggregate, and show sharper declines at lower temperatures. Rice, which both sustains lower damage at high temperatures^14^, and is relatively scarcely cultivated in Sub-Saharan Africa^59^, shows a comparatively weaker relationship that is flat and noisy at high temperatures.

**Figure 4:**
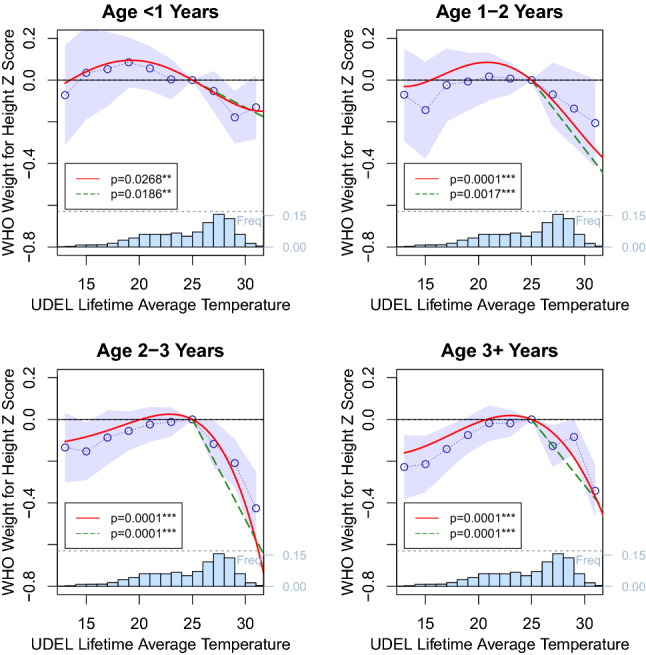
Lifetime average temperature and child weight-for-height by age group. Weight-for-height is plotted against the average temperature over all months of a child’s life, relative to a lifetime average temperature of 25 $^{\circ }$C (histogram at base of plot). Three model results are shown: a binned model in blue with confidence intervals, a fourth order polynomial in red and a degree-month model in green. All models include region, interview year, and interview month fixed effects, as well as controls for precipitation and child and mother demographics. *p* values for the polynomial (joint significance of polynomial terms) and degree-month model are shown.

Finally, we estimate the effect of lifetime average temperatures on weight-for-height in Fig. 4 (weight-for-age, height-for-age, and disaggregated rural and urban results are shown in Fig. S11–S16). We find that child weight declines with lifetime average temperatures exceeding 25 $^{\circ }$C, and intensifies with age. We find a mild negative result for children under 1, consistent with potentially confounding fetal selection and infant mortality effects, stronger results among 1–2 year olds, and strongest effects for children over 2 years old. For these children, shifting lifetime average temperature from 25 to 30 $^{\circ }$C is associated with a roughly 0.5$\sigma$ decrease in weight-for-height, equivalent in magnitude to the 0.1$\sigma$ per 1 $^{\circ }$C gradient seen in the cross-section.

**Figure 5:**
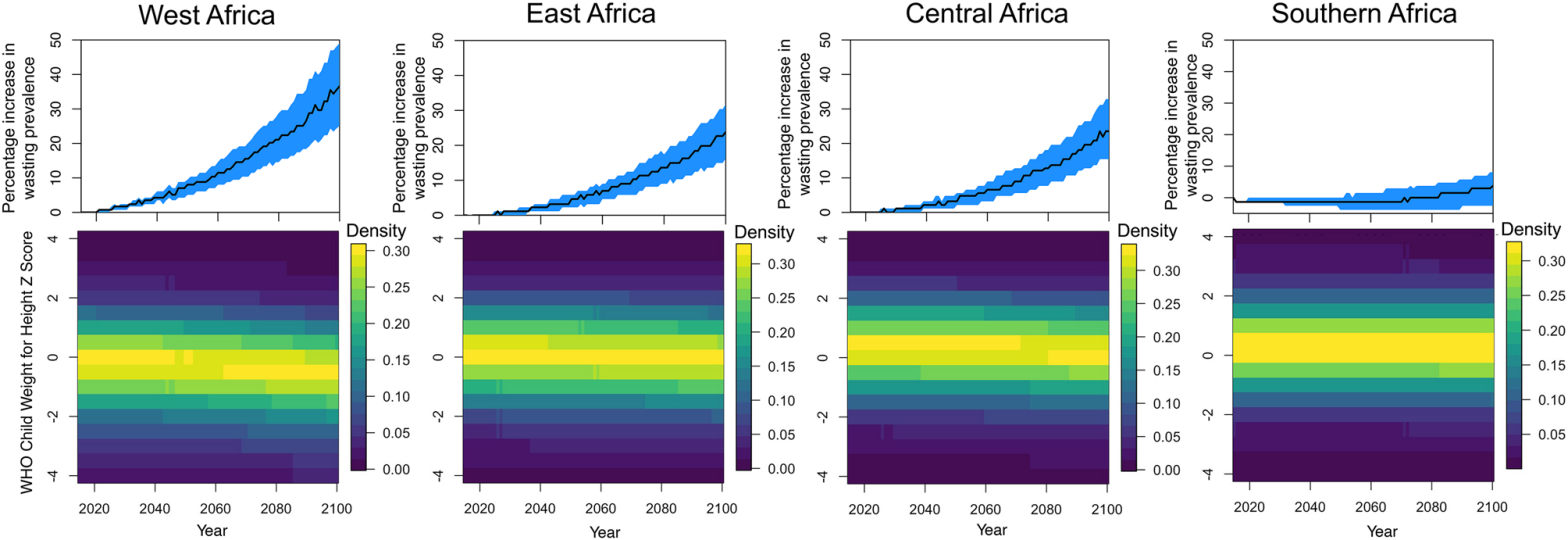
Implications of future temperature changes for child wasting. Top row shows the percentage increase in the prevalence of wasting, relative to 2015 proportions, for each region. 95% confidence intervals, based on parameter uncertainty, are shown in blue. Bottom row surface plots show the predicted distributions of child weight-of-age for all years from 2015 to 2100. Temperature projections are taken from CMIP5 multi-model mean based on a RCP8.5 emissions scenario.

Climate change will increase temperatures in Sub-Saharan Africa over the coming century, with the region expected to be one of the more severely affected^3^ (Fig. S25). These high temperatures are expected to lead to significant losses in agricultural yields if adaptation does not take place^11^. Figure 5 provides projections for the effect of future warming on child weight-for-height and wasting, relative to 2015, based on a RCP8.5 emissions scenario and temperature projection data from the CMIP5 multi-model mean. Because of the uncertainty in rainfall projections for the region, and because precipitation was not a significant driver in our model, we focus only on mean temperature projections^56^. Projections are based on a combined model for rural and urban locations (Fig. S6, “Methods”).

We find that future effect of warming on child-wasting may be very severe depending on location. In western Africa temperatures are already frequently above the 25 $^{\circ }$C tipping point predicted by our model. As the climate warms the region experiences more of the very high temperatures we find to be crucially detrimental to child weight, resulting in predictions for a 37% increase in the proportion of children affected by wasting by 2100. In contrast, milder temperatures in some parts of southern Africa pose less of a threat to child wasting in the present. Future warming, on average in this region, does not bring these temperatures above the threshold, so wasting percentages stay broadly constant. For east Africa and central Africa climate change results in a 25% increase in wasting prevalence.

## Discussion

Our results outline a robust association between high ambient temperatures and child weight loss in both cross-sectional population averages as well as dynamically, across different time scales. The largest weight loss is seen in rural areas, where the effect appears to be driven by a loss of agricultural yields caused by excess heating during growing season^60^. The effects we observe in urban locations are smaller and shorter-lived, consistent with the well-documented link between temperature and diarrhea. Pooling the whole sample, we estimate lifetime effects of temperature on weight-for-height and -for-age close to the 0.1$\sigma$ per C$^{\circ }$ decrease observed in the cross-section, implying that a substantial portion of the gradient in child weight observed across regions could be attributable to temperature.

There are several caveats to our results. While our climate dataset provides rich variation across space and time, our child dataset is limited in that children are only observed once. This means our research design cannot be used to directly determine the long-term implications of temperature-driven child weight on adult outcomes or mortality, nor can we explicitly disentangle mechanistic pathways driving the temperature-growth link over the course of a child’s life. In particular, data tracking individuals over long time periods could prove useful for understanding the implications of high temperatures for chronic malnutrition, which we do not observe in our results.

A second limitation is that we match temperature data based on the child’s present location, however, the child may have moved from another location during the exposure period. For 46% of our sample, information was available on the length of time resident in the current location. We found our results were broadly robust to running our main regressions on the subset of children reported as resident for the exposure period (Fig. S4, S5), though these results are noisier due to the reduced sample. Third, we focus on monthly average temperatures which may ignore important temperature fluctuations at the daily level. Results using daily temperatures are shown in Fig. S19–S21 and are similar to our monthly temperature results. Fourth, our climate change projections do not include the effect of precipitation. Given the relationship between precipitation and agricultural yields, it is likely changes to precipitation will affect nutrition outcomes in the future. We chose to exclude precipitation because our regression results did not show a clear and consistent effect (Fig. S18), however, exploring the link between precipitation and child nutrition is an important avenue for future work. Finally, while our results provide an *average effect* of temperature on child anthropometric outcomes, there is likely significant heterogeneity across individuals. Individual economic circumstances, as well as distinct local cropping practices, could result in highly varied outcomes for each individual child.

Our climate change projections suggest that malnutrition will increase markedly over the coming decades in certain locations, with western Africa being a particular region of concern. These results do not take into account other economic, demographic and technological changes that could alter child development pathways. While adaptation and policy choices, such as improved urban sanitation infrastructure and rainfall insurance, could improve future prospects, population growth and migration may put increased pressure on limited resources. Urbanization may interact with these drivers in a complex way: our results suggest that urban households are more insulated from the long-run impacts of high temperature exposure, yet it is unclear how much of this effect is driven by economic circumstances, shown also to be important (Fig. S8). Regardless, our results quantify an additional hurdle regions may face in improving child growth metrics, one that will need to be accounted for in pursuing the Sustainable Development Goals for reducing undernutrition.

## Methods

### Data

We use data taken from the Demographic and Health surveys (DHS) (https://dhsprogram.com/), a publicly available comprehensive survey dataset. The DHS maintains strict ethical standards; DHS survey methods are approved by the ICF Institutional Review Board (IRB) as well as equivalent boards within host countries. All survey information is gathered in accordance with the guidelines and regulations of these institutions. Informed consent was given by all respondents and by parents or guardians for children under 18. Further details of DHS ethical procedures are available at https://dhsprogram.com/What-We-Do/Protecting-the-Privacy-of-DHS-Survey-Respondents.cfm. We take all surveys available that contain child anthropometric data as well as GPS-tagged survey locations. After removing potential erroneous measurements in the survey our dataset contains approximately 280,000 observations from 28 countries (Fig. S1) and 190,000 children over the age of 1.

The World Health Organization measures growth standards for children with three variables: weight-for-height, child weight-for-age and child height-for-age. A child’s measurement for any of these variables can be represented as a Z-Score on a distribution where the mean is the expected value of child growth in normal environmental conditions. All anthropometric variables used in this study are calculated using the WHO Anthro software (https://www.who.int/childgrowth/software/en/) to generate standardized growth measures (see Fig. 1 in the main text). The units used in this case are standard deviations ($\sigma$) of the normalized distribution. We remove any flagged observations where weight-for-height, weight-for-age or height-for-age exceeded +/− 6$\sigma$ per WHO guidelines to remove potentially erroneous observations.

We use GPS coordinates from the DHS survey to assign each child to a 0.5 * 0.5$^{\circ }$ latitude-longitude gridcell in our climate dataset. We use climate data from the University of Delaware’s gridded station-based dataset [Willmott, C. J. and K. Matsuura (2001) Terrestrial Air Temperature and Precipitation: Monthly and Annual Time Series (1950–1999)]. The data is provided at a 0.5 * 0.5$^{\circ }$ spatial resolution and a monthly temporal resolution. For climate change projections we use the CMIP5 multi-model mean based on the RCP8.5 emissions scenario, accessed via the KNMI Climate Explorer (https://climexp.knmi.nl/start.cgi). For population projections we use country-level projections from the World Bank.

### Regression model

We examine the relationship between temperature and child growth using several different approaches. We first estimate the average relationship in the cross section, which provides descriptive population-level evidence though it confounds factors such as poverty and ecology which may be correlated with both temperature and child growth. To ameliorate these concerns, we then estimate a standard fixed-effects ordinary least squares regression model of temperature on child anthropometric outcomes, controlling for both spatially and temporally invariant factors to exploit conditionally exogenous variation in temperature over the period preceding surveys^5,6,22^. Our approach is conceptually similar to comparing children surveyed within the same subnational region of a country at different times, and hence with different temperature exposures, while also removing the average sample effect of being surveyed at that specific time, and within that specific region.

The main regression equation is a ordinary least squares regression model of the form:

1 $$\begin{aligned} A_{i,r,c,m,y} = \nu T_{i,m=0} + \beta f(T_{i,y}) + \gamma \varvec{X}_{i} + \omega _{c,y} + \delta _{r} + \mu _{m} + \epsilon _{i,c,y} \end{aligned}$$

where $A_{i,r,c,m,y}$ is an anthropometric measurement from child *i* interviewed in region *r* located in country *c* during month *m* of year *y*. Temperature for annual and lifetime exposure is calculated by one of three different functional forms $f(T_{i,y})$: a binned count model of monthly temperatures over the relevant time period with average monthly temperatures from 24–26 °C being the omitted count, a degree-month model for monthly temperatures exceeding 25 $^{\circ }$C and a fourth order polynomial model in monthly temperature over the previous year. These three models can be directly compared and are based on standard approaches to revealing non-linear temperature effects^6^. $\nu T_{m=0}$ is temperature in the month of interview, which acts as a control for the annual level regression. $\varvec{X}_{i}$ is a vector of child-specific controls, including mother’s education, mother’s age and child birth order interacted with child sex, birth month as well as precipitation in the month of interview as well as cumulative precipitation over the previous year. Birth month is included in our controls as it is likely correlated with seasonal drivers^61^, though recall issues have been found for this variable^62^. In general, recall issues on timing of birth may add noise to our lifetime exposure findings.

In order to remove spatial forms of bias we include indicator variables for each sub-national region, $\delta _{r}$ (admin level 1), such that average spatial differences at the subnational scale are accounted for. These regions were identified in the DHS data and standardized across survey waves, aggregating up in cases where region boundaries changed over time. To account for temporal bias we include indicator variables for each country-by-year survey observation, $\omega _{c,y}$, capturing average country-level trends in anthropometrics, as well as a calendar month-of-interview indicator variable $\mu _{m}$ to control for arbitrary seasonal effects in the sample. As such, the identifying assumption is that for children surveyed within a region, changes in recent temperature are uncorrelated with other trending variables that affect weight and height. We include a vector of controls plausibly unrelated to temperature changes at the time scales we observe, including mother’s age, child birth month, education and child birth-order interacted with sex. Robustness checks for this model, including different fixed effect specifications, are shown in Table S1. Errors, $\epsilon _{i,c,y}$, are clustered at the country-by-year level and sample is reweighted using DHS survey weights in all regression models.

Regressions are run with the same nonlinear temperature functions, controls and fixed effects for the month of survey, year before survey and lifetime average temperature of each child. We limit our sample to include children over the age of 1 year to minimize selection and scarring effects associated with in-utero heating exposure known to cause changes in fertility, miscarriage, and infant mortality, effects for which we provide evidence using estimates of temperature exposure by age^40,42,43^.

We use monthly temperature due to the limited availability daily station-based data in this region. Daily data based on interpolated satellite data is however available^63^. Results using this data source are shown in Section S6 of the supplement. The results support the effect size of the main result when scaled, however, cannot be directly compared as the daily data is based on daily maximum values and the monthly UDEL is based on the mean.

We also test for the robustness of our results when including the sub-sample of women who report being resident in their location for the previous year, or lifetime of the child. This question was not included in many of the DHS survey waves and was only asked to 46% of our sample. Of this sample, 88% report being resident in their current location for longer than 1 year and 85% report being resident for the entire lifetime of the child. Our results are robust to evaluating temperature effects for this subset (Fig.  S4–S5).

### Crop season effects

We use a high resolution (approximately 10km by 10km resolution at the equator^59^) gridded crop dataset to identify the effect of growing season temperature on child weight-for-height. The dataset provides information on multiple crops grown in each location including the area given over to each crop and the length of the growing season.

We merge the child dataset with the crop dataset using latitude and longitude coordinates. We then subset the dataset by locations that grow maize, wheat or rice, noting that many locations grow several or all of these crops. We then calculate temperature measures (binned, polynomial and degree-month) during the growing season for each crop. Finally we run a weighted regression of growing season temperature on child weight-for-height for each crop where weights are the proportion of the local area given over to a particular crop. This means that locations that grow solely one crop type will contribute more to the identification of the crop growing season effect than regions growing a much smaller proportion of the same crop. This allows us to separately identify growing season effects for different crops even though many locations grow multiple different crops. Results from these regressions are shown in Fig. 3.

### Future wasting

We use Eq. (1), the annual regression, to forecast the future effect of climate change on child wasting. For each country we take projections from the CMIP5 multi-model mean for each month from 2015 to 2100. We use the polynomial version of Eq. (1), based on combined rural and urban locations (Fig. S6), to calculate the effect of temperature changes on child weight loss relative to 2015 such that 2015 represents the currently observed distribution. We apply these changes to the distribution of children in each of four regions as found in our data. We assume that children across the distribution experience the same weight loss and do not take into account disparate effects by quantile. Mean projected changes to temperature in each region are shown in Fig. S25.

### Selection tests

Height-for-age shows a slight positive relationship with lifetime temperature (Fig. S15). Evidence of improving health with worsening environmental conditions is often taken as evidence of selection on treatment and sample attrition in the early child and in-utero health literature^37,40,64^. High temperatures are known to increase mortality in many contexts, and mortality risk is high with weight decline, such that children with a Z-Score below − 3$\sigma$ have an approximately 30–50% case-fatality rate^65^. We test for evidence of early life selection by first removing all children under age 1 from the sample and re-estimating lifetime temperature effects on height-for-age; doing so removes the positive association and leaves marginal effects flat (Fig. S17). We further investigate heating effects in-utero and find that hot temperatures later in pregnancy decrease both children’s weight and height metrics at time of interview, consistent with heating scarring children and reducing growth (Fig. S22–S24). Heating during the first trimester, when the risk of heat-induced fertility reductions and miscarriage to selectively alter sample composition is highest^9,42,43^, are found to increase height-for-age (Fig. S23), suggesting that sample attrition is confounding results and selecting for healthier infants in the cohort, consistent with similar findings in Sub-Saharan African adult women^42^.

## Supplementary information

Supplementary Information.

## Data Availability

All data used in this study are publicly available. Links to data sources are provided in the methods.
