# Supplementary material for: Characterizing the contribution of high temperatures to child undernourishment in Sub-Saharan Africa
Source: Sci Rep. 2020 Nov 2;10:18796. doi: 10.1038/s41598-020-74942-9 (PMC7606522; doi:10.1038/s41598-020-74942-9)
Supplement: Supplementary file 1 — Supplementary Information. [file 41598_2020_74942_MOESM1_ESM.pdf]

Supplementary Materials

Characterizing the contribution of high temperatures to child undernourishment in Sub-Saharan Africa

Rachel E. Baker and Jesse Anttila-Hughes

Contents

S1 Data **2**

S2 Robustness of main model **5**

S3 Whole sample results for Figure 2 **9**

S4 Lifetime temperature **12**
- S4.1 Weight-for-height . . . 12
- S4.2 Weight-for-age . . . 14
- S4.3 Height-for-age . . . 16

S5 Precipitation **19**

S6 Daily temperature data **20**
- S6.1 Weight for Height . . . 20
- S6.2 Weight for Age . . . 20
- S6.3 Height for Age . . . 21

S7 In-utero effect **21**

S8 Climate change projection **23**

S1 Data

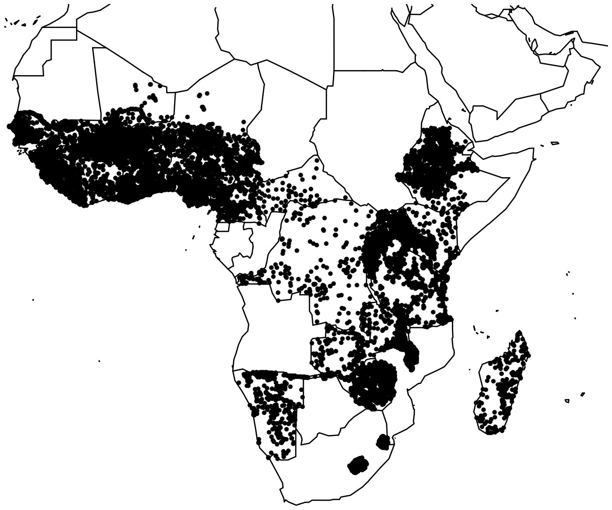

Figure S1: A map showing the locations of all DHS clusters used in this study.

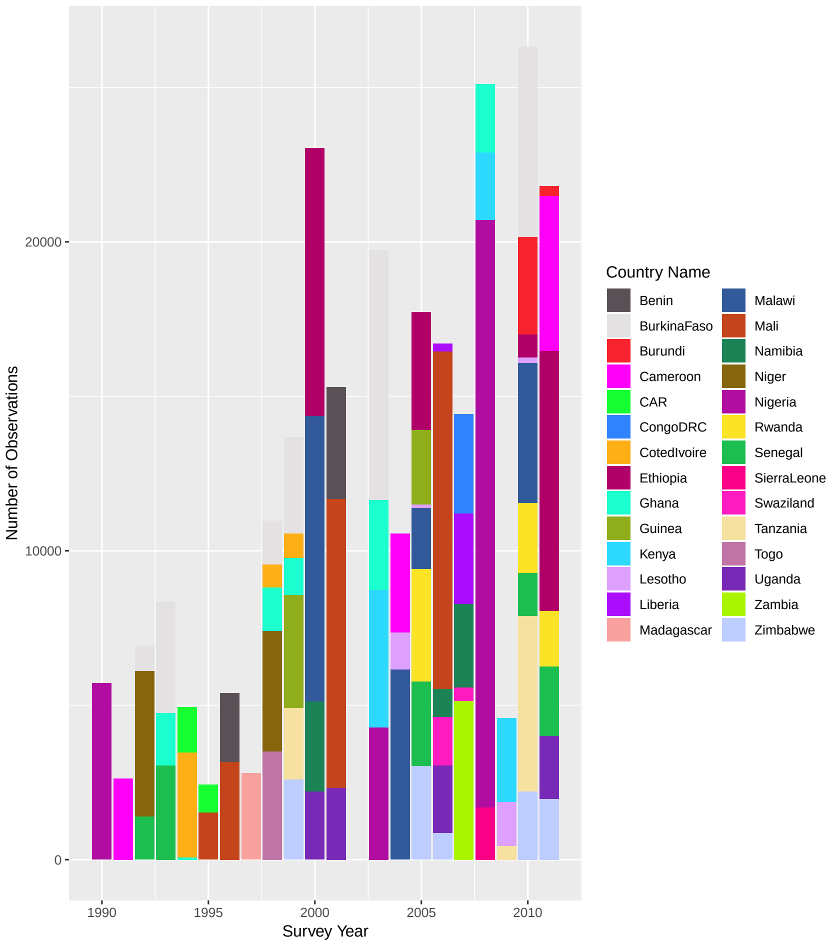

Figure S2: A bar chart shows the distribution of the data in terms of country and survey year where units are number of child-level observations.

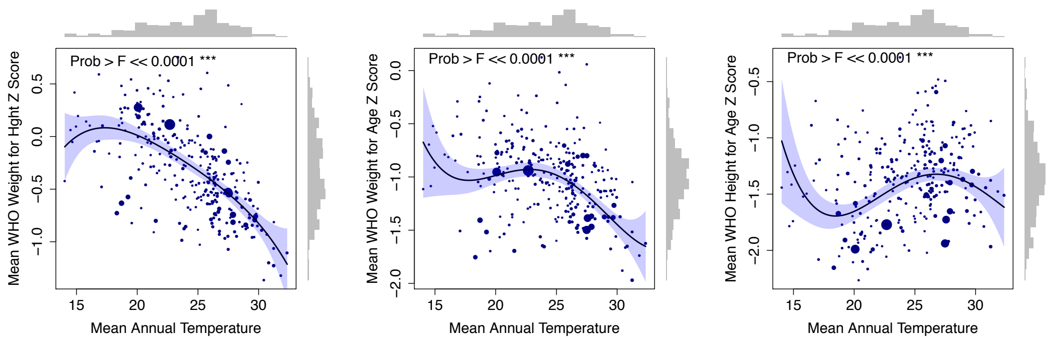

Figure S3: Sub-national regional correlation between average (year before survey) regional temperature and average anthropometric measures for children surveyed in that region. A fourth-order polynomial is fitted to the data, weighted by number of observations (proportional to dot size). The F-statistic for the polynomial fit is shown along with histograms of the distribution of the data.

S2 Robustness of main model

	Dependent variable:					
	Weight-for-Height Z Score					
	(1)	(2)	(3)	(4)	(5)	(6)
Degree Month > 25C	-0.0091^{***}	-0.0092^{***}	-0.0070^{***}	-0.0067^{***}	-0.0069^{***}	-0.0067^{***}
	(0.00165)	(0.00167)	(0.0020)	(0.0019)	(0.0019)	(0.0006)
Mother Secondary Education				0.131^{***}	0.131^{***}	0.131^{***}
				(0.017)	(0.017)	(0.012)
Mother Higher Education				0.336^{***}	0.334^{***}	0.336^{***}
				(0.037)	(0.037)	(0.032)
Female				0.017	0.018	0.017
				(0.017)	(0.017)	(0.016)
Mother's Age at Birth				0.022^{***}	0.023^{***}	0.022^{***}
				(0.005)	(0.005)	(0.005)
Mother's Age at Birth ^{2}				-0.0004^{***}	-0.0004^{***}	-0.0004^{***}
				(0.0001)	(0.0001)	(0.0001)
Controls	No	No	No	Yes	Yes	Yes
Fixed Effects 1	Country	Country*Year	Country*Year	Country*Year	Country*Year	Country*Year
Fixed Effects 2	Year	Month	Month	Month	Country*Month	Month
Fixed Effects 3	NA	NA	Region	Region	Region	Region
Std Error Cluster	Country*Year	Country*Year	Country*Year	Country*Year	Country*Year	Household
Observations	192,362	192,362	192,362	192,362	192,362	192,362
R-squared	0.074	0.076	0.090	0.092	0.094	0.092

*p<0.1; **p<0.05; ***p<0.01

Table S1: Robustness checks for the regression of temperature on child weight-for-height using the degree-month function form. Model 4 is reported in the paper. Controls also include temperature in the month-of-interview, birth order*gender and precipitation, not included due to space constraints.

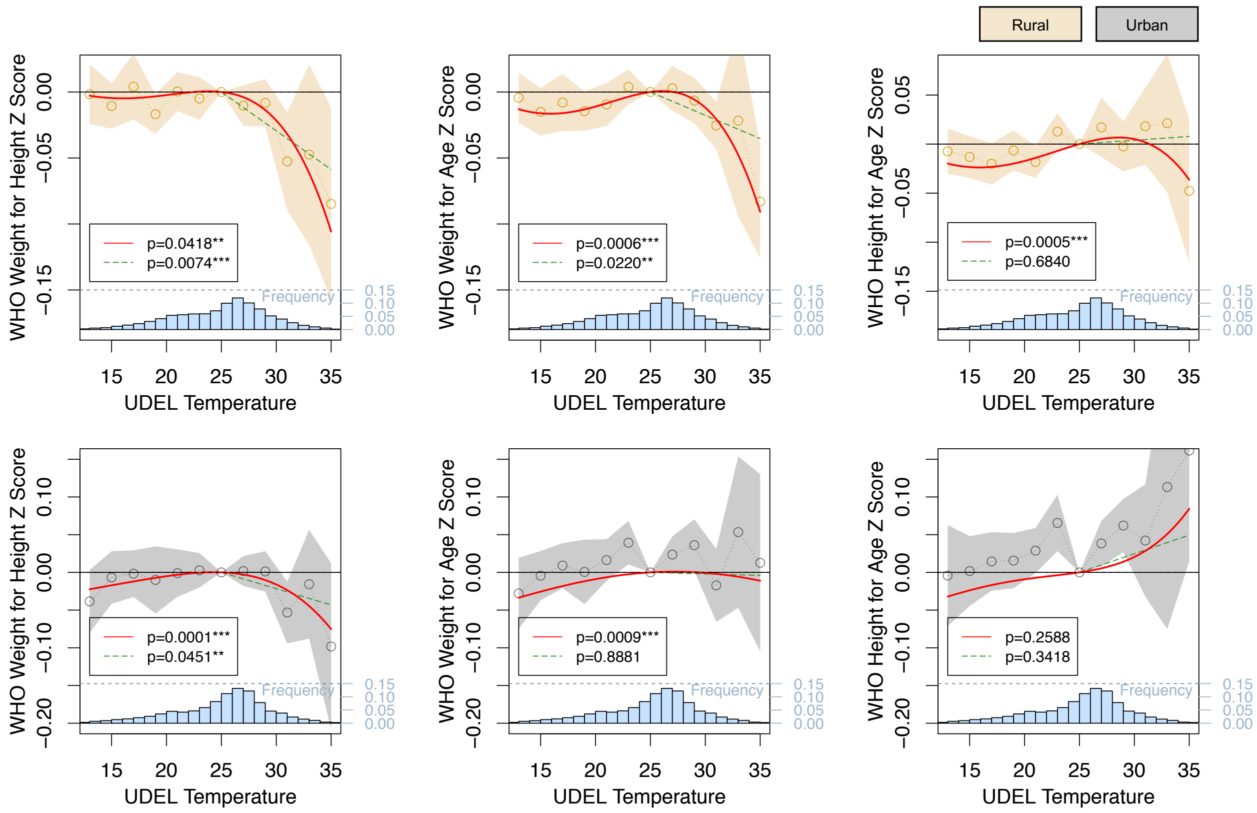

Figure S4: The main result, as plotted in Figure 2A, using only the sub-sample of women who report being resident in their current location for more than a year. This controls for potential selection effects due to migration. This question was only asked in certain survey waves, such that the sample with non-NA answers to this question is 46% of our original sample. Of this sub-sample, 88% of women report being resident for longer than 1 year.

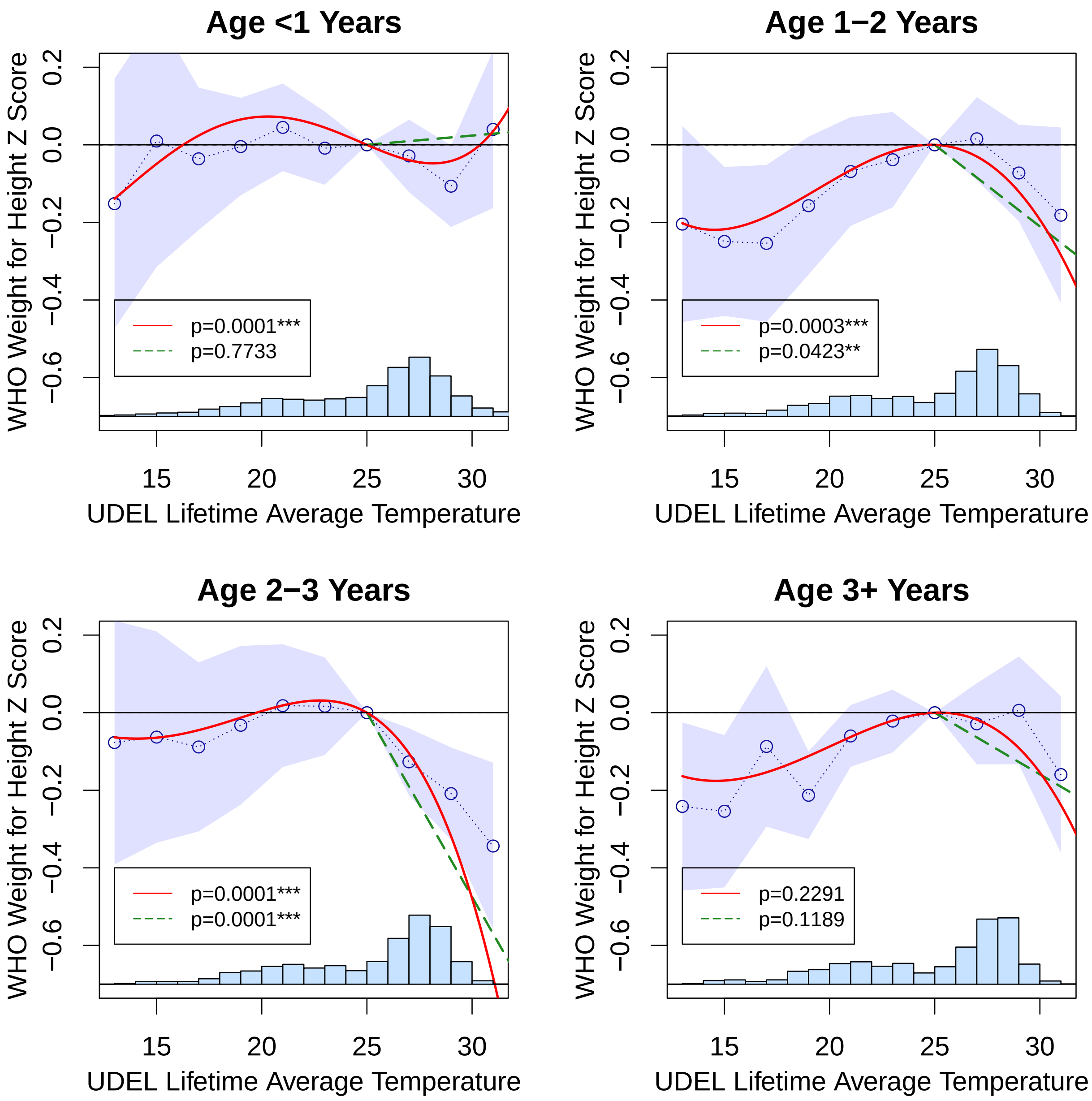

Figure S5: The main result, as plotted in Figure 4, using only the sub-sample of women who report being resident in their current location for the length of the child lifetime. This controls for potential selection effects due to migration. This question was only asked in certain survey waves, such that the sample with non-NA answers to this question is 46% of our original sample. Of this sub-sample, 85% of women report being resident for the child's lifetime.

	Dependent variable:					
	Weight-for-Height Z Score		Weight-for-Age Z Score		Height-for-Age Z Score	
	(1)	(2)	(3)	(4)	(5)	(6)
Degree Month > 25C	−0.0072***		−0.0038***		0.0028	
	(0.0019)		(0.0014)		(0.0185)	
Degree Month > 25C *T1		−0.0052		−0.0048		−0.0024
		(0.0036)		(0.0046)		(0.0044)
Degree Month > 25C *T2		−0.0080***		−0.0054**		0.0004
		(0.0021)		(0.0022)		(0.0030)
Degree Month > 25C *T3		−0.0040**		−0.0022		0.0009
		(0.0017)		(0.0025)		(0.0035)
Degree Month > 25C *T4		−0.0088***		−0.0037**		0.0044**
		(0.0024)		(0.0016)		(0.0018)
Controls	Yes	Yes	Yes	Yes	Yes	Yes
Fixed Effects 1	Country*Year	Country*Year	Country*Year	Country*Year	Country*Year	Country*Year
Fixed Effects 2	Year	Month	Month	Month	Month	Month
Fixed Effects 3	Region	Region	Region	Region	Region	Region
Note:						*p<0.1; **p<0.05; ***p<0.01

Table S2: Robustness checks for the regression of temperature on child outcomes over different time periods, using the rural-area sample, using the degree-month function form. All main model controls are included. The degree-month variable is interacted with an indicator for different time periods separated into 5-year bins where T1 is 1990-1995, T2 is 1995-2000, T3 is 2000-2005, and T4 is $\geq$ 2005.

S3 Whole sample results for Figure 2

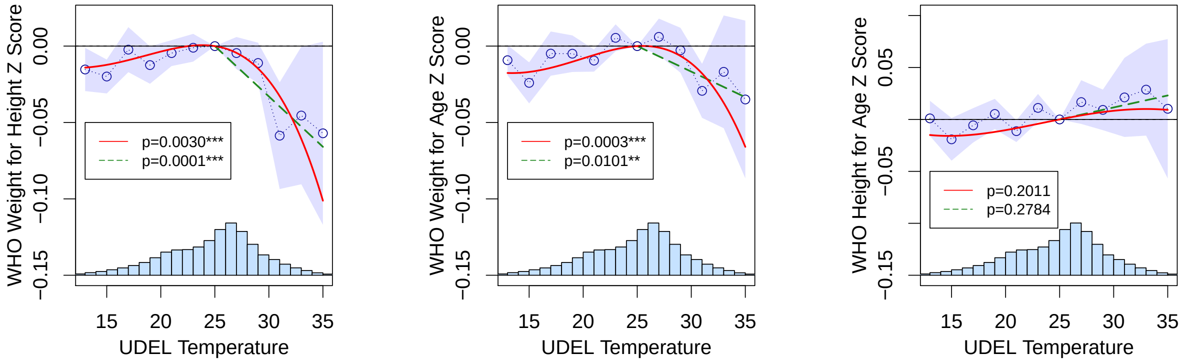

Figure S6: The main result, as plotted in Figure 2A for the whole sample in our dataset. P-values for the polynomial (joint significance of polynomial terms) and degree-month model are shown.

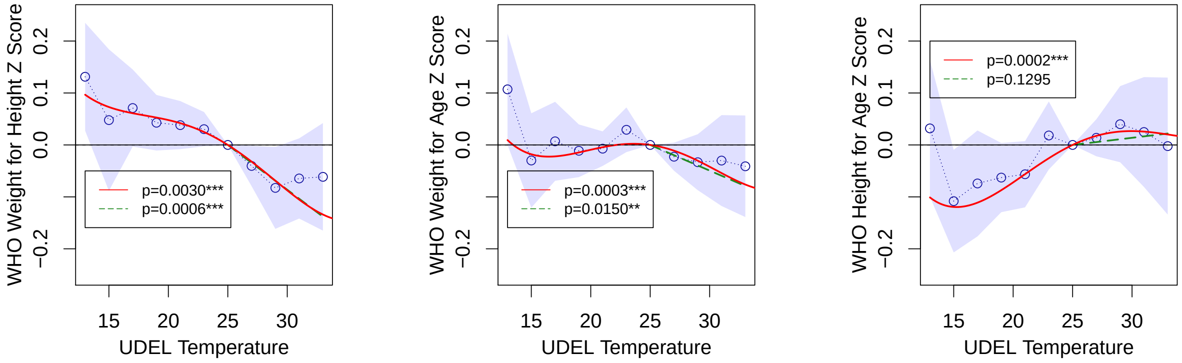

Figure S7: The main result, as plotted in Figure 2B for the whole sample in our dataset. P-values for the polynomial (joint significance of polynomial terms) and degree-month model are shown.

Sub-samples

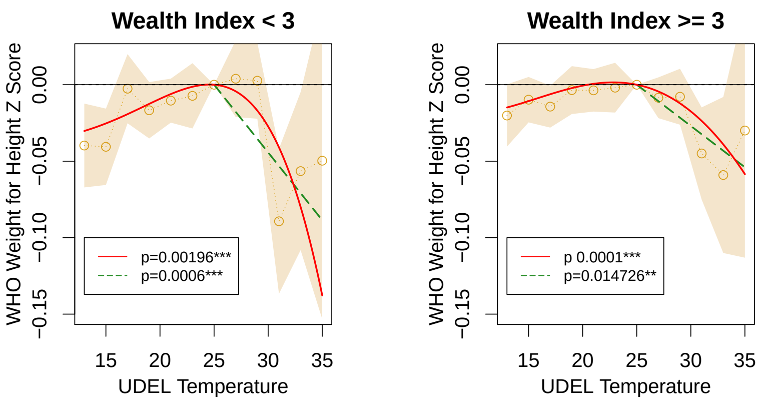

Figure S8: The effect of temperature over the previous year on child weight-for-height for rural households with different wealth indices. The wealth index provides a measure for wealth divided into five quintiles. We split the sample to look at lower 40% of wealth versus upper 60%. Both divisions appear to show a response of weight to temperatures. The response is steeper among the lower quintiles. P-values for the polynomial (joint significance of polynomial terms) and degree-month model are shown.

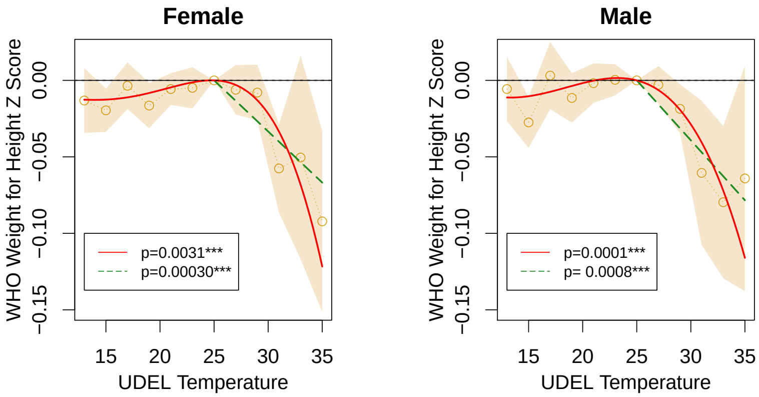

Figure S9: The effect of temperature over the previous year on child weight-for-height by gender in rural locations. P-values for the polynomial (joint significance of polynomial terms) and degree-month model are shown.

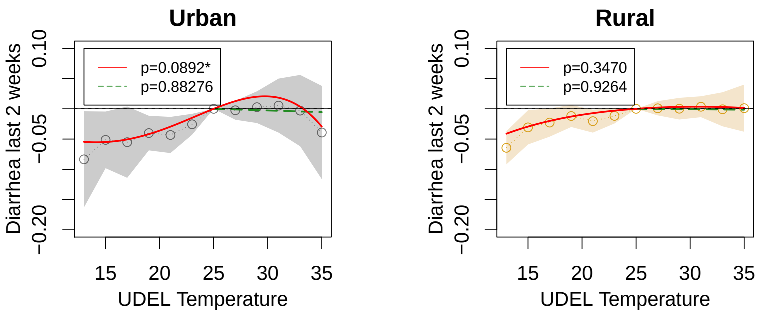

Figure S10: The effect of temperature in the previous month on the probability a child is reported as having diarrhea in the last two weeks. Urban response and rural response is shown separately. P-values for the polynomial (joint significance of polynomial terms) and degree-month model are shown.

S4 Lifetime temperature

S4.1 Weight-for-height

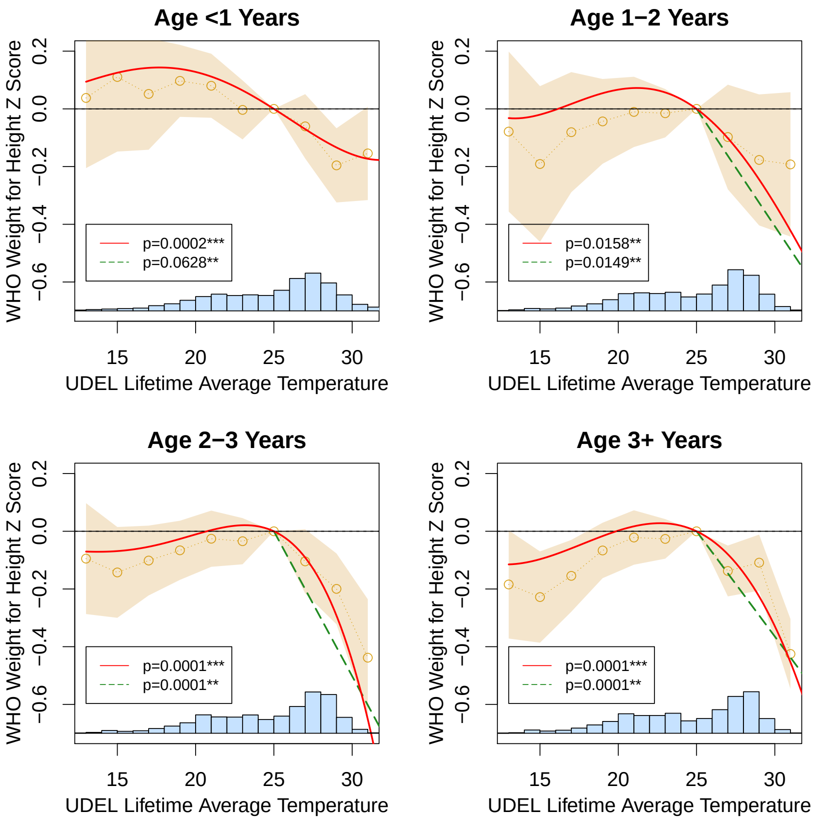

Figure S11: The effect of lifetime-averaged temperature on child weight-for-height, by age group, for rural locations. P-values for the polynomial (joint significance of polynomial terms) and degree-month model are shown.

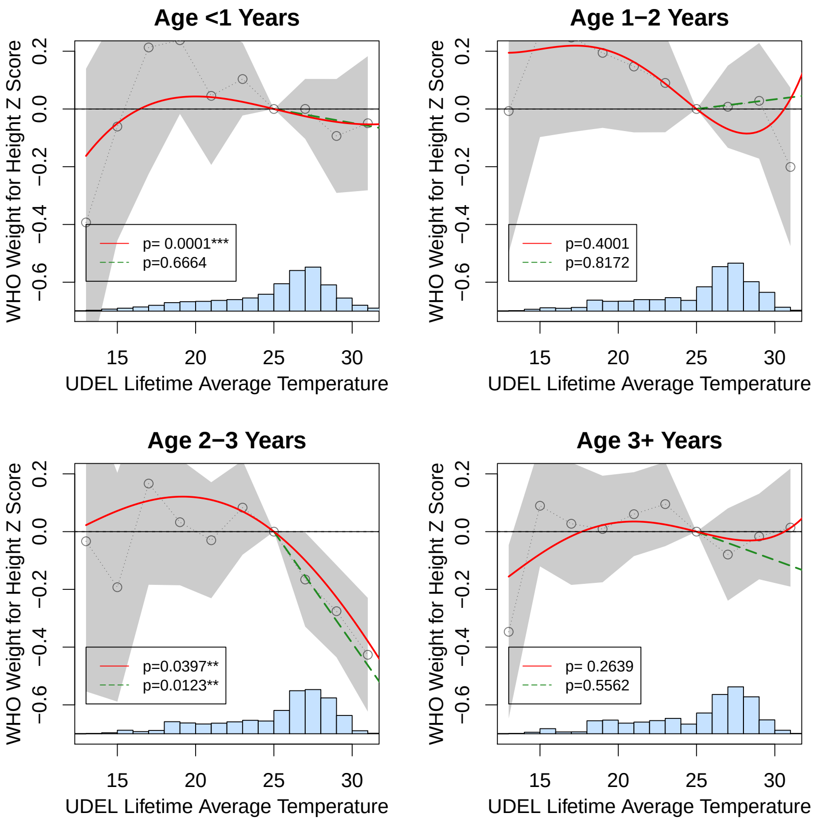

Figure S12: The effect of lifetime-averaged temperature on child weight-for-height, by age group, for urban locations. P-values for the polynomial (joint significance of polynomial terms) and degree-month model are shown.

S4.2 Weight-for-age

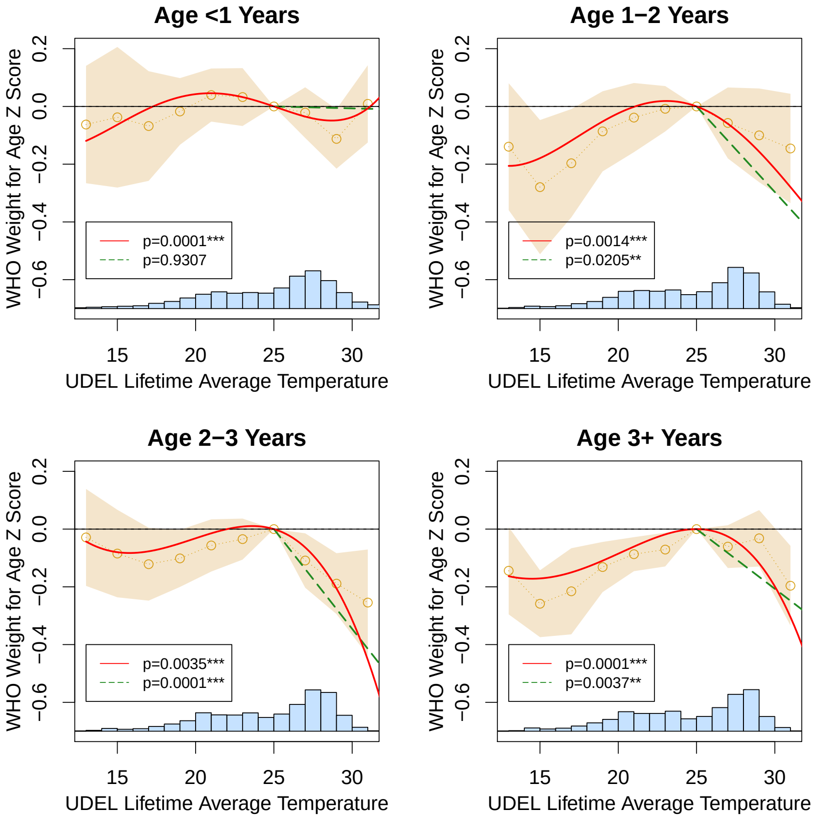

Figure S13: The effect of lifetime-averaged temperature on child weight-for-age, by age group, for rural locations. P-values for the polynomial (joint significance of polynomial terms) and degree-month model are shown.

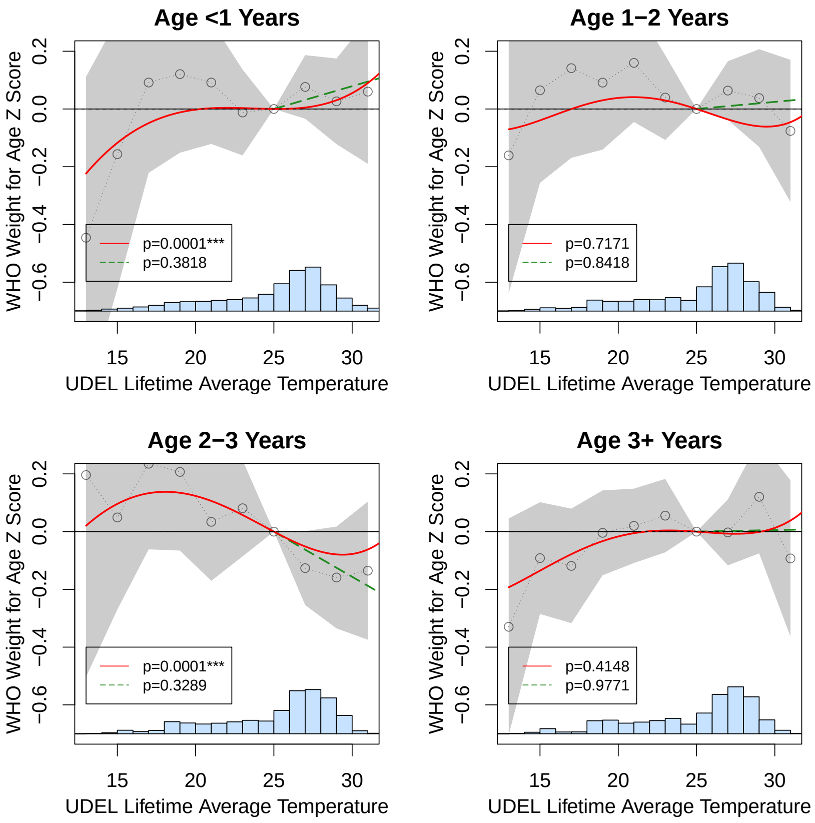

Figure S14: The effect of lifetime-averaged temperature on child weight-for-age, by age group, for urban locations. P-values for the polynomial (joint significance of polynomial terms) and degree-month model are shown.

S4.3 Height-for-age

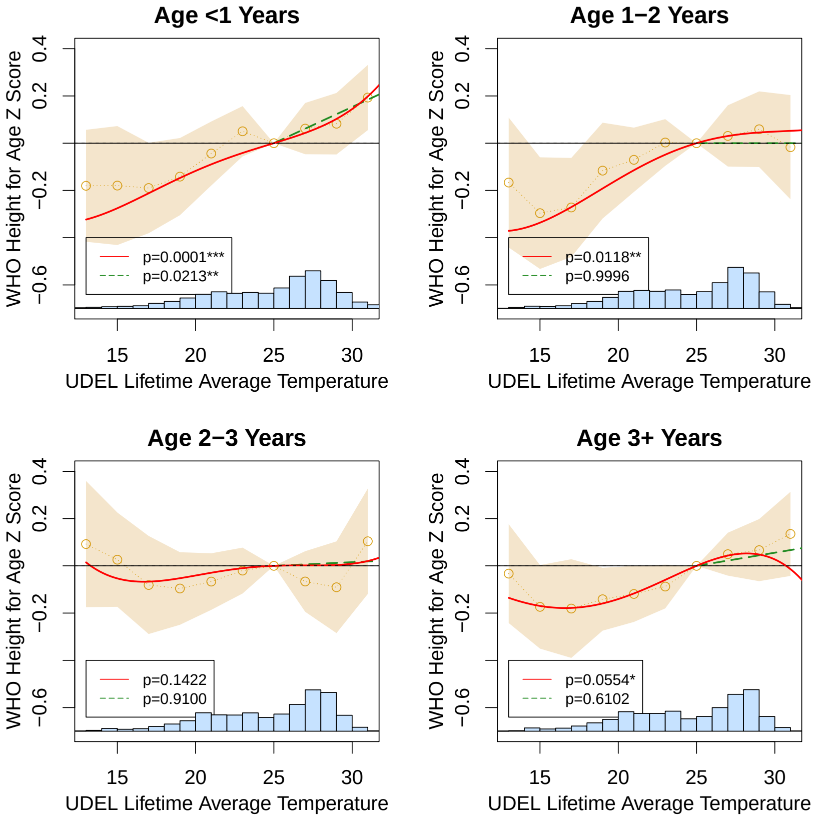

Figure S15: The effect of lifetime-averaged temperature on child height-for-age, by age group, for rural locations. P-values for the polynomial (joint significance of polynomial terms) and degree-month model are shown.

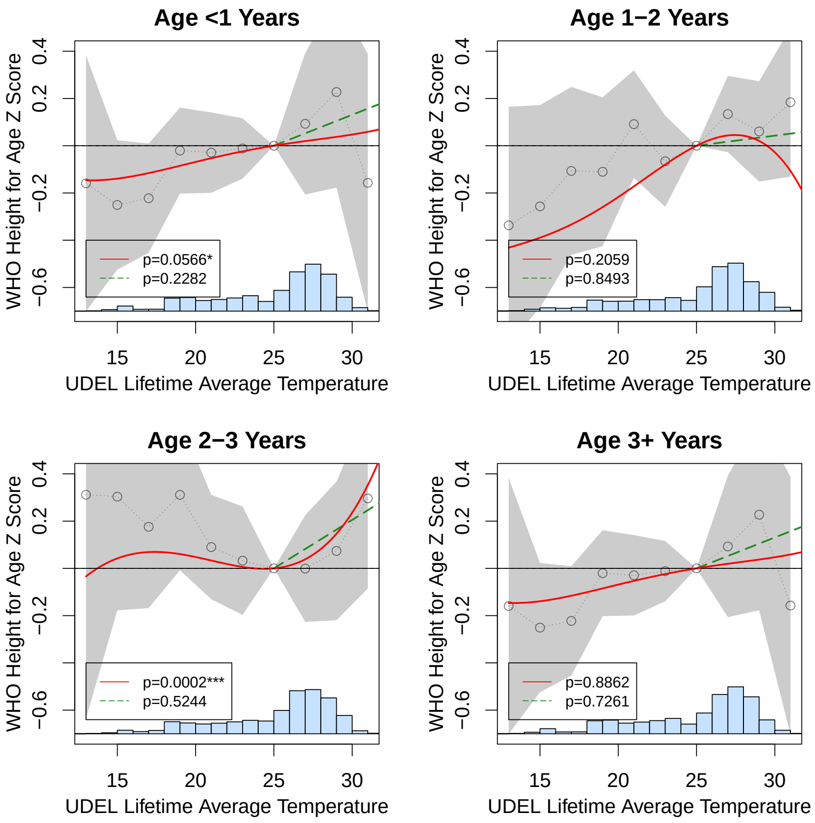

Figure S16: The effect of lifetime-averaged temperature on child height-for-age, by age group, for urban locations. P-values for the polynomial (joint significance of polynomial terms) and degree-month model are shown.

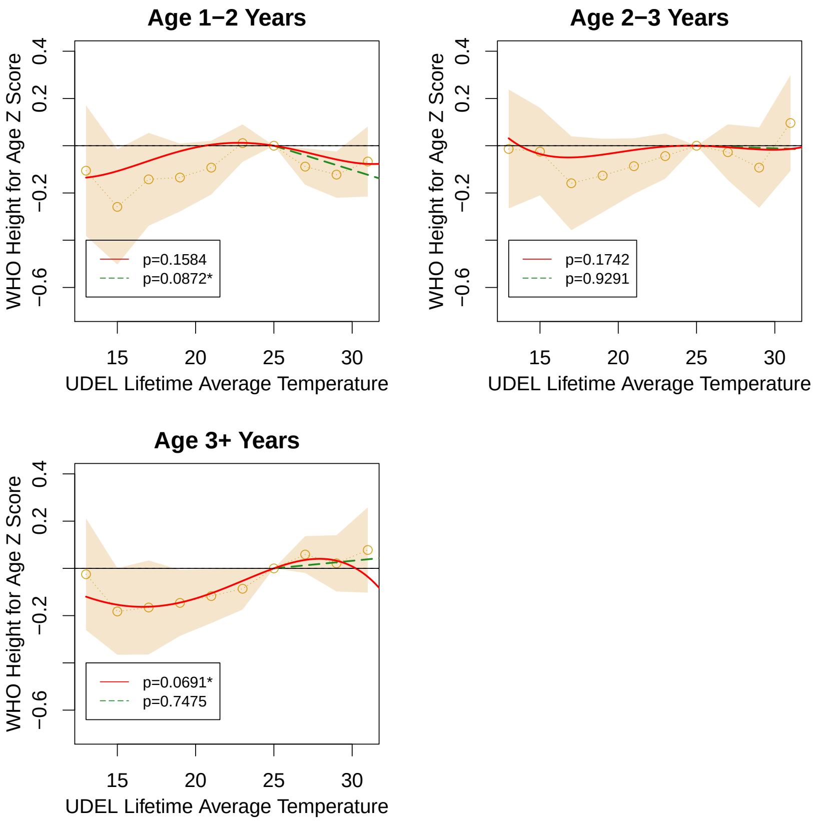

Figure S17: We test for evidence of early life selection by first removing all children under age 1 from the sample and re-estimating lifetime temperature effects on height-for-age; doing so removes the positive association and leaves marginal effects flat. P-values for the polynomial (joint significance of polynomial terms) and degree-month model are shown.

S5 Precipitation

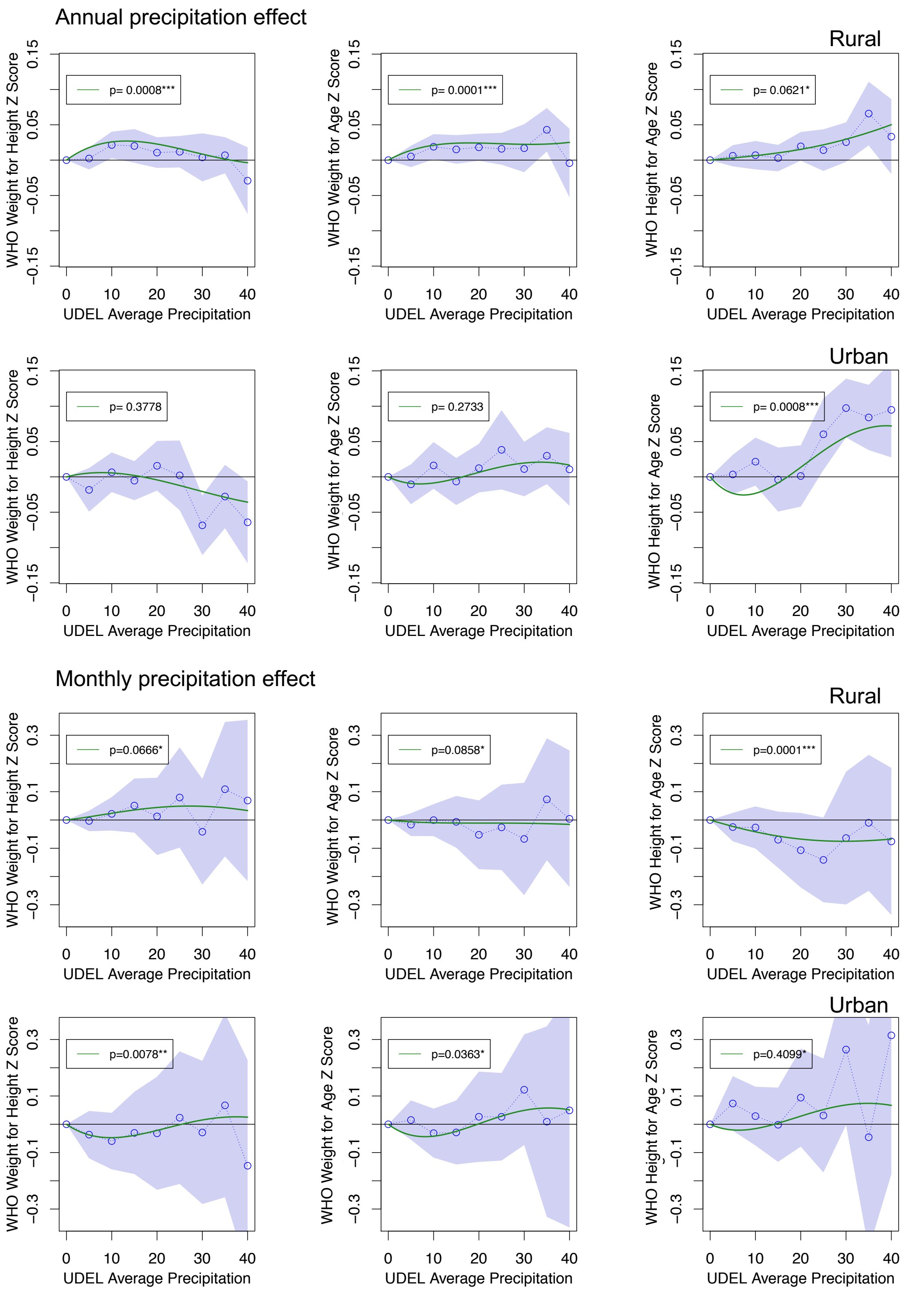

Figure S18: The binned effect of precipitation, in the previous year on the three anthropometric variables. The model controls for temperature as well as the suite of demographic controls and the same fixed effects as in equation (1). P-values for the polynomial (joint significance of polynomial terms) are shown.

S6 Daily temperature data

S6.1 Weight for Height

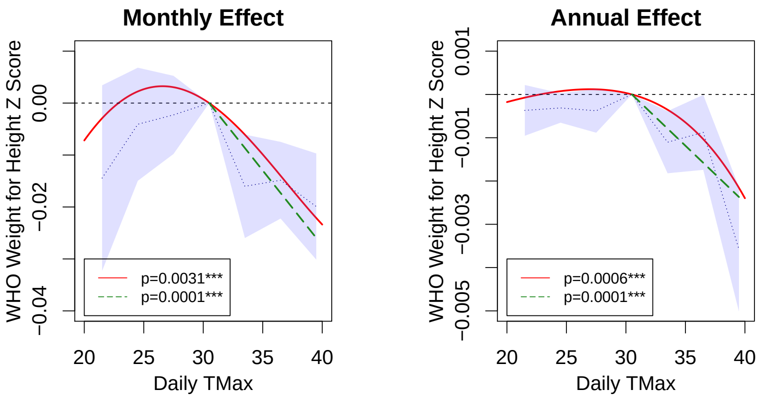

Figure S19: The effect of daily maximum temperature, using data from the Princeton African Flood and Drought Monitor, on WHO weight for height calculated using days in the previous month (right) and year (left). Controls are the same as the main specification. Values are of similar magnitude to monthly effects when scaled. Daily temperature data in this region relies on temporal interpolation and therefore are autocorrelated. P-values for the polynomial (joint significance of polynomial terms) and degree-month model are shown.

S6.2 Weight for Age

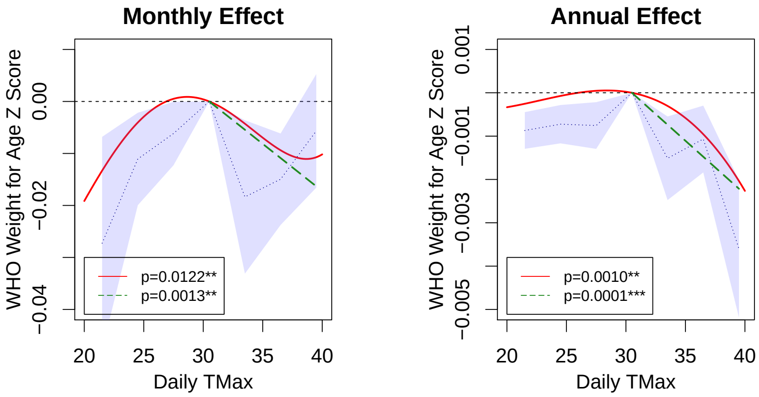

Figure S20: The effect of daily maximum temperature, using data from the Princeton African Flood and Drought Monitor, on WHO weight for age calculated using days in the previous month (right) and year (left). P-values for the polynomial (joint significance of polynomial terms) and degree-month model are shown.

S6.3 Height for Age

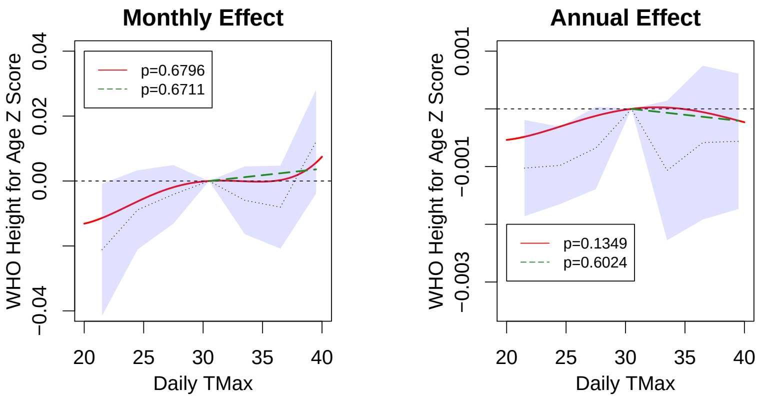

Figure S21: The effect of daily maximum temperature, using data from the Princeton African Flood and Drought Monitor, on WHO height for age calculated using days in the previous month (right) and year (left). P-values for the polynomial (joint significance of polynomial terms) and degree-month model are shown.

S7 In-utero effect

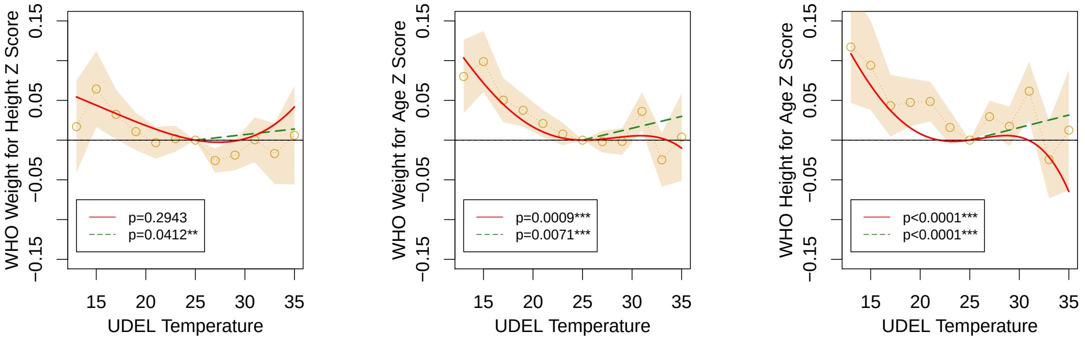

Figure S22: The effect of temperature in utero on later life anthropometric measures. This model includes mother fixed effects to control for household level covariates. P-values for the polynomial (joint significance of polynomial terms) and degree-month model are shown.

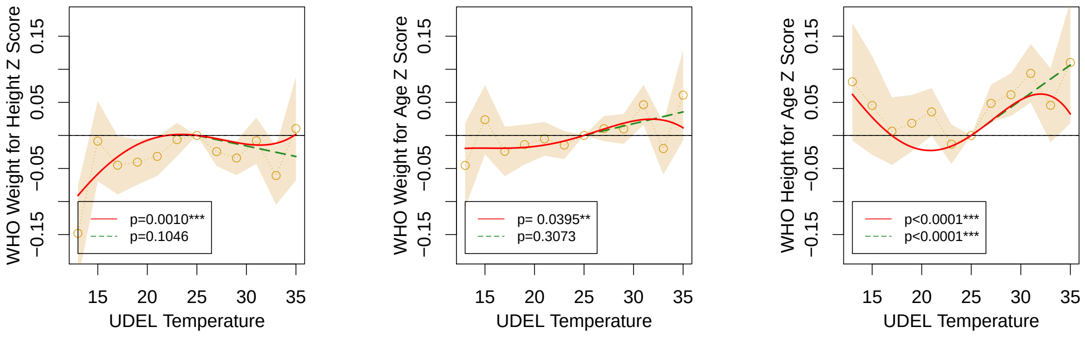

Figure S23: The effect of temperature in utero, in the first trimester only, on later life anthropometric measures. This model includes mother fixed effects to control for household level covariates. Heating during the first trimester, when the risk of heat-induced fertility reductions and miscarriage to selectively alter sample composition is highest, are found to increase height-for-age, suggesting that sample attrition is confounding results and selecting for healthier babies in cohort. P-values for the polynomial (joint significance of polynomial terms) and degree-month model are shown.

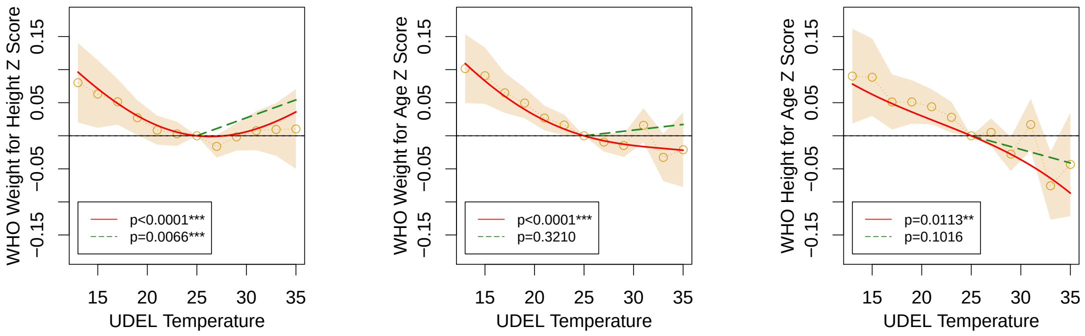

Figure S24: The effect of temperature in utero, in the second and third trimesters, on later life anthropometric measures. This model includes mother fixed effects to control for household level covariates. P-values for the polynomial (joint significance of polynomial terms) and degree-month model are shown.

S8 Climate change projection

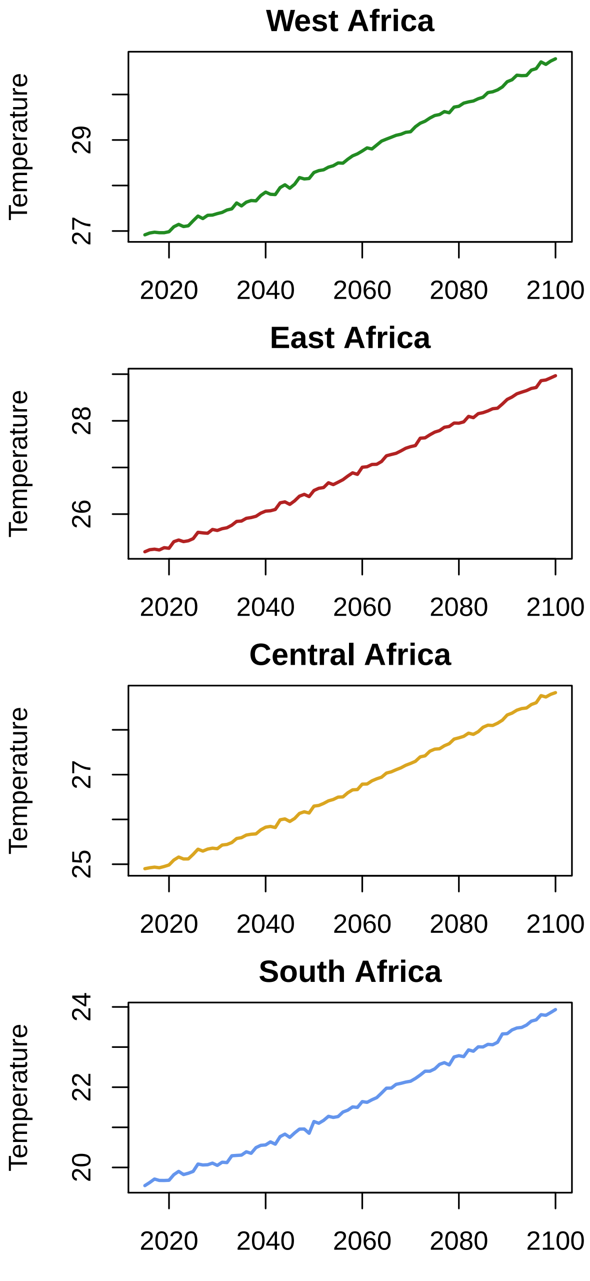

Figure S25: Projected average temperature changes for four regions of sub-Saharan Africa based on the CMIP5 multi-model mean scenario RCP8.5.

Region	Country	In Sample
Central Africa	Angola	
Central Africa	Burundi	X
Central Africa	Cameroon	X
Central Africa	Central African Republic	X
Central Africa	Chad	
Central Africa	Congo-Brazzaville	
Central Africa	Democratic Republic of Congo	X
Central Africa	Gabon	
Central Africa	Rwanda	X
East Africa	Djibouti	
East Africa	Eritrea	
East Africa	Ethiopia	X
East Africa	Kenya	X
East Africa	Madagascar	X
East Africa	Malawi	X
East Africa	Mozambique	
East Africa	Somalia	
East Africa	Tanzania	X
East Africa	Uganda	X
Southern Africa	Botswana	
Southern Africa	Lesotho	X
Southern Africa	Namibia	X
Southern Africa	South Africa	
Southern Africa	Swaziland	X
Southern Africa	Zambia	X
Southern Africa	Zimbabwe	X
West Africa	Benin	X
West Africa	Burkina Faso	X
West Africa	Cote d'Ivoire	X
West Africa	Ghana	X
West Africa	Guinea	X
West Africa	Guinea-Bissau	
West Africa	Liberia	X
West Africa	Mali	X
West Africa	Mauritania	
West Africa	Niger	X
West Africa	Nigeria	X
West Africa	Senegal	X
West Africa	Sierra Leone	X
West Africa	Togo	X
West Africa	Western Sahara	

Table S3: The countries that we use to make future projections by region. Countries in our dataset are shown in the third column.
